# Evaluating Rutting Resistance of Rejuvenated Recycled Hot-Mix Asphalt Mixtures Using Different Types of Recycling Agents

**DOI:** 10.3390/ma15248769

**Published:** 2022-12-08

**Authors:** Tameem Mohammed Hashim, Mohammed Salah Nasr, Yasir Mohammed Jebur, Abdullah Kadhim, Zainab Alkhafaji, Mirza Ghouse Baig, Saheed Kolawole Adekunle, Mohammed A. Al-Osta, Shamsad Ahmad, Zaher Mundher Yaseen

**Affiliations:** 1 Department of Building and Construction Techniques Engineering, Al-Mustaqbal University College, Hillah 51001, Iraq; 2 Technical Institute of Babylon, Al-Furat Al-Awsat Technical University (ATU), Najaf 51015, Iraq; 3 Civil and Environmental Engineering Department, King Fahd University of Petroleum & Minerals, Dhahran 31261, Saudi Arabia; 4 Interdisciplinary Research Center for Construction and Building Materials, King Fahd University of Petroleum and Minerals, Dhahran 31261, Saudi Arabia

**Keywords:** HMA, RAP, rejuvenators, waste recycling, rutting index

## Abstract

Growing environmental pollution worldwide is mostly caused by the accumulation of different types of liquid and solid wastes. Therefore, policies in developed countries seek to support the concept of waste recycling due to its significant impact on the environmental footprint. Hot-mix asphalt mixtures (HMA) with reclaimed asphalt pavement (RAP) have shown great performance under rutting. However, incorporating a high percentage of RAP (>25%) is a challenging issue due to the increased stiffness of the resulting mixture. The stiffness problem is resolved by employing different types of commercial and noncommercial rejuvenators. In this study, three types of noncommercial rejuvenators (waste cooking oil (WCO), waste engine oil (WEO), and date seed oil (DSO)) were used, in addition to one type of commercial rejuvenator. Three percentages of RAP (20%, 40%, and 60%) were utilized. Mixing proportions for the noncommercial additives were set as 0–10% for mixtures with 20% RAP, 12.5–17.5% for mixtures with 40% RAP, and 17.5–20% for mixtures with 60% RAP. In addition, mixing proportions for the commercial additive were set as 0.5–1.0% for mixtures with 20% RAP, 1.0–1.5% for mixtures with 40% RAP, and 1.5–2.0% for mixtures with 60% RAP. The rutting performance of the generated mixtures was indicated first by using the rutting index (G*/sin δ) for the combined binders and then evaluated using the Hamburg wheel-track test. The results showed that the rejuvenated mixtures with the commercial additive at 20 and 60% RAP performed well compared to the control mixture, whereas the rejuvenated ones at 40% RAP performed well with noncommercial additives in comparison to the control mixture. Furthermore, the optimum percentages for each type of the used additives were obtained, depending on their respective performance, as 10%, 12.5%, and 17.5% of WCO, 10%, 12.5–17.5%, and 17.5% of WEO, <10%, 12.5%, and 17.5% of DSO, and 0.5–1.0%, 1.0%, and 1.5–2.0% of the commercial rejuvenator, corresponding to the three adopted percentages of RAP.

## 1. Introduction

### 1.1. Research Background

Pavements, especially asphaltic pavements, are reusable. The pavement’s service life depends on essential factors, mainly the weight and volume of traffic, construction quality, subgrade strength, drainage system, weather, and quality of construction materials [1,2]. The service life of any pavement can be prolonged by employing timely maintenance [3,4]. Ultimately, pavement degradation sets in, causing failure of the pavement. Old extracted materials from the pavement can be recycled and reused in reconstruction [5,6]. Reclaimed asphalt pavement (RAP) is a valuable, high-quality material that can be used instead of expensive new aggregates and asphalt binders [7].

On average, 95% of asphalt pavement is made up of aggregate (coarse, fine, and filler) and 5% binder (bitumen), which is the asphalt mixture’s most expensive variable component [8]. Most of the time, asphalt binder is used in the top and middle layers of flexible pavement to protect the asphalt pavement structure and subgrade from moisture [9]. Thus, asphalt binder aids in constructing a smooth and skid-resistant riding surface that can withstand the wear caused by traffic and has enough tensile strength to keep the pavement safe from warping [5]. Furthermore, RAP’s best use is in flexible pavements where a portion of the expensive virgin binder can be replaced with a cheaper RAP binder in the surface and intermediate course [6]. The advantages of utilizing RAP include its environmental friendliness, affordability, and excellent bonding properties, whereas its disadvantages relate to its color and quality [6].

Using RAP in new asphalt mixtures will rejuvenate the old RAP binder and become a part of the adhesive that sticks the mixture’s components together. Similarly, that old aggregate will be a part of the total aggregate content in the mix [10,11]. At the same time, the amount of virgin aggregate used in the asphalt mixtures can be reduced by utilizing RAP. Additionally, using RAP reduces the cost and amount of new binder (bitumen) required in the construction process of asphalt pavement [12].

The American National Cooperative Highway Research Program (NCHRP) 2001 developed a Superpave mix design protocol for handling RAP as a technician manual (NCHRP-452, 2001) for managing high RAP recycled mixtures [13]. In 2017, The American Association of State Highway and Transportation (AASHTO) came up with the AASHTO M 323 specifications for Superpave volumetric mix design [14]. These instructions for choosing the binder grade for RAP mixes were mostly based on the assumption that RAP binder and virgin binder would be mixed completely through specific blending charts [15].

The aged RAP binder is very stiff and has a high viscosity, causing different types of failure in the mixtures, such as low workability and low-temperature cracking [16]. Many studies have examined this issue and tried to solve it using different approaches, including blending with a softer binder, adopting the warm-mix asphalt (WMA) technique, using a foamed binder, and using recycling agents. 

Due to the high percentage of volatile components in their chemical composition, bio-based oils such as fatty soy acids are extremely vulnerable to aging. They could be used as a fluxing agent for improving recycled asphalt’s properties. Waste cooking oil (WCO) or vegetable oil has a flash point greater than 220 °C, making it suitable for hot asphalt mixtures [17]. As a result of the cooking process at high temperatures, the volatile components of waste cooking oil are significantly reduced, making it less susceptible to aging. The amount of asphalting in an asphalt binder can be reduced by mixing it with waste cooking oil based on the Fourier-transform infrared spectroscopy (FTIR) test [17]. Waste engine oils (WEO) are recycling agents utilized to revitalize aged RAP binders to the specified limits. By incorporating a small amount of WEO into recycled mixtures, it creates pavement with performance comparable to that of pavement constructed entirely from virgin materials. It was reported that WEO can lower the stiffness of recycled mixtures and soften the virgin binder without affecting the moisture sensitivity of the pavement [18].

All the mentioned rejuvenators (WCO, WEO, and DSO) have noncommercial origins and can be provided through different sources, such as public restaurants, car maintenance stations, and local farms. SonneWarmix RT™ is a paraffinic hydrocarbon wax used as a commercial recycling agent/rejuvenator, especially for the HMA mixtures containing RAP, and can be sourced from the local markets. Previous studies recommended using this wax as a recycling agent because it increases the rutting resistance of the mix [19].

### 1.2. Literature Review

Recent studies have examined the use of date seed oil (DSO) as a rejuvenator for recycled HMA mixtures. It has been reported that using DSO as a recycling agent increased the fatigue resistance of mixtures containing 20% RAP. However, it deteriorated the mixture’s resistance to rutting failure [20]. Mamun et al. [21] investigated the use of waste engines and cooking oils as rejuvenators in recycled HMA mixtures. Based on their results, using 7% of WEO at 40% RAP achieved better performance, while using 13% of WCO at 50% RAP also achieved better results. On the other hand, Al-Saffar et al. [22] examined the performance and durability of rejuvenated asphalt mixtures incorporating various rejuvenators (including WCO and DSO). Their results provide a key benchmark for predicting future issues revitalizing aging asphalt. Furthermore, Moghaddam et al. [23] conducted a review study on using RAP in hot-mix asphalt (HMA) mixtures and rejuvenating the stiff combined mixtures using different recycling agents, including WCO, WEO, and SonneWarmix RT^TM^.

Yin et al. [24] investigated the effectiveness of long-term rejuvenation on binder blends and mixtures containing a high RAP content. The results indicated that the rejuvenating blend of aged binders had restored all the rheological properties, producing a high cracking resistance pavement. In addition, O’Sullivan et al. [25] presented a laboratory study on recycling aged RAP binders using rejuvenators. All the rejuvenated HMA mixtures were tested for dynamic modulus. The study recommended the vital use of rejuvenators within HMA-RAP mixtures.

The two primary factors that cause fracture in asphalt pavement, particularly rutting, are temperature and stress brought on by the load (permanent deformation). Therefore, many measures have been taken, such as enhancing pavement quality and structural design approaches, to lessen the issues with the rutting of roadways. In recent years, many engineers have been more focused on modifying and improving the performance of asphalt by adding various additives and substituting recycled materials for virgin asphalt to enhance the environment and lower the price of modified pavement mixtures. In their study, Joni and AL-Rubaie [26] examined the effects of adding low-density waste polyethylene to asphalt mixes in percentages of 2, 4, and 6% by weight of asphalt and their effects on how well the mixtures function at extreme temperatures. This research demonstrated that the performance of asphalt mixes at various high temperatures was enhanced using plastic waste (low-density polyethylene) as a bitumen modifier. This was accomplished by employing low-density polyethylene waste at an ideal amount of roughly 4% by weight of asphalt and improving the Marshall stability by utilizing this proportion of polymer. The rut depth was reduced by 80.5% and 82.3% at temperatures of 50 and 60-degree centigrade, respectively.

### 1.3. Research Motivation

Sustainable development in several sectors has encouraged using waste materials as a raw material substitution. Because of the size of road networks, it is possible to employ waste from many sectors in road pavement to save money and protect the environment, which has sparked substantial studies [27,28]. Recycling agents can soften or rejuvenate the aged binder, reducing its stiffness and cracking potential by introducing some of the aged asphalt’s lost components. Virgin binders can be used if the RAP binder has not deteriorated significantly [29]. As noted, pavement sustainability can be improved by including waste materials in the procedures [30].

Because of the axial force of moving cars and the large increase in traffic volume over the last several years, the service life of road pavement has greatly diminished. Hot mixture asphalts (HMA) often fail because of permanent deformation, which is typical in tropical areas. The properties of the primary components, namely bitumen and aggregates, significantly impact the performance and strength of HMA against various failures [31]. However, the current research addresses using recycled materials and finding recycled materials with low rutting stress.

### 1.4. Research Objectives

This study investigated three noncommercial rejuvenators (WCO, WEO, and DSO) and one commercial rejuvenator in asphaltic mixtures incorporating RAP at three levels (20%, 40%, and 60%). All the asphaltic mixtures were prepared and evaluated under the Superpave design method. In contrast, the elected aggregate gradation for preparing the mixtures is for a surface course mixed with 20%, 40%, and 60% RAP. The novelty of this study is presented by comparing the performance of commercial and noncommercial rejuvenators utilized in recycled HMA mixtures to produce a sustainable high-performance mixture capable of withstanding significant rutting stresses. All the developed Superpave mixtures are evaluated in terms of volumetric properties (air voids, voids filled with asphalt, voids of mineral aggregate) and rutting resistance using the Hamburg-wheel track test to achieve an optimal sustainable rejuvenated recycled HMA mixture with a high percentage of RAP capable of exhibiting comparable or better performance to the virgin HMA mixture. 

## 2. Experimental Program

### 2.1. Materials

#### 2.1.1. Reclaimed Asphalt Pavement (RAP)

**Figure 1:**
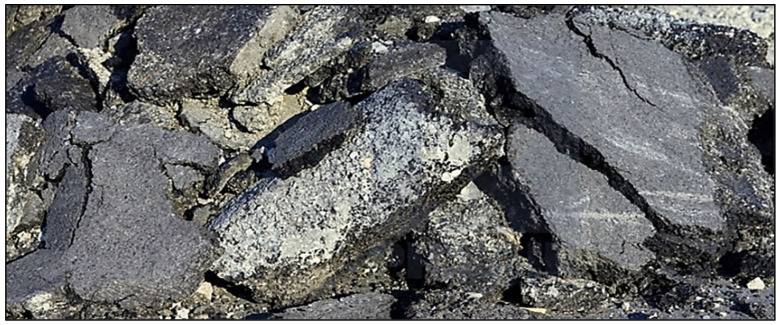
RAP stockpile.

The RAP used in this study was collected from an old stockpile of asphalt pavement waste, as shown in Figure 1, which has been scraped for a surface layer. The gathered RAP was then crushed, screened, and stockpiled. The properties of the processed RAP are presented in Table 1. Using the AASHTO T 308 ignition method, the binder content of RAP was recovered. In contrast, the gradation of RAP was obtained according to AASHTO T 27 [23] for the recovered aggregate, as shown in Table 2.

#### 2.1.2. Aggregate

This study used the normal crushed coarse aggregate of limestone origin from a quarry in Al-Najaf city. In contrast, the fine aggregate was brought from Karbala city; both are cities in Iraq. To achieve the Iraqi standards for coarse surface gradation (SCRB, R/9) [32], coarse and fine aggregates are sieved and then recombined in a certain ratio. The combined aggregate gradation is selected for the surface layer, as shown in Table 3. 

On the whole, routine tests were carried out to assess the physical features of the combined aggregate. The results and the SCRB’s specified limits are summarized in Table 4.

#### 2.1.3. Asphalt Binder

Al Nasiriyah (40–50) penetration grade binder was employed. The breakdown of the binder’s physical characteristics is shown in Table 5.

#### 2.1.4. Mineral Filler

This study used ordinary Portland cement as a filler, as in Table 6.

#### 2.1.5. Noncommercial Rejuvenators

- Waste Cooking Oil (WCO)

**Figure 2:**
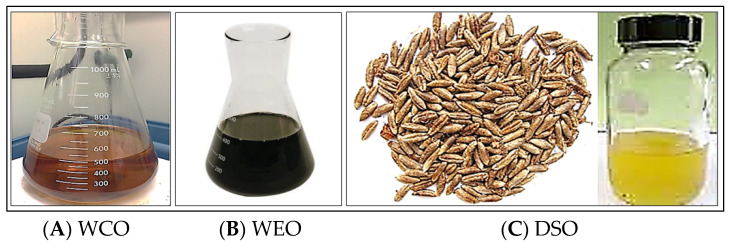
Samples of the used noncommercial additives.

This study used waste cooking oil gathered from residential places as a recycling agent/rejuvenator, as shown in Figure 2A. Most of the cooking oil’s structure was made up of fatty acids [33]. Its physical characteristics and chemical components are presented in Table 7 and Table 8.

- Waste Engine Oil (WEO)

As shown in Figure 2B, the waste oil used for gasoline vehicles is produced mainly from paraffinic oils, with a small amount of added chemicals to enhance its viscosity, stability, cleanliness, and inflammability. As a lubricant, WEO may also comprise short-chain polar molecules that disassemble simultaneously [34]. Its physical characteristics are presented in Table 9.

- Date Seed Oil (DSO)

This oil was extracted directly from the seeds of dates, as shown in Figure 2C, forming about 15% of its weight. Oleic acid is the main component of the oil, forming about 50% of its structure. Using the DSO as an efficient rejuvenator reduces such material’s environmental footprint and minimizes the pavement’s total cost [20]. Its physical characteristics and chemical components are presented in Table 10 and Table 11.

#### 2.1.6. Commercial Rejuvenator

**Figure 3:**
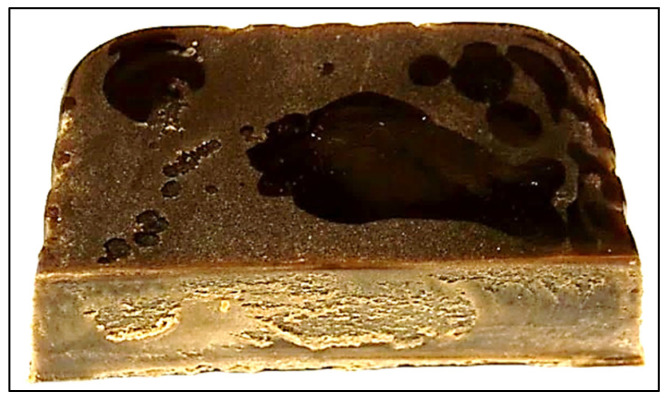
SonneWarmix RT^TM^ sample.

The commercial rejuvenator used in the study was SonneWarmix RT™. It is a paraffinic wax produced by Sonneborn as an additive for asphalt mixtures. Due to its performance in terms of increasing the workability of the recycled mixture and reducing the inhalation emissions, it has been considered a promising recycling agent/rejuvenator, in addition to its compatibility with the RAP. The manufacturer’s recommended dosage is 0.5–2.0% by weight of the binder [35]. A sample of SonneWarmix RT™ is shown in Figure 3, and its physical characteristics are presented in Table 12.

### 2.2. Design of Asphalt Mixtures

**Figure 4:**
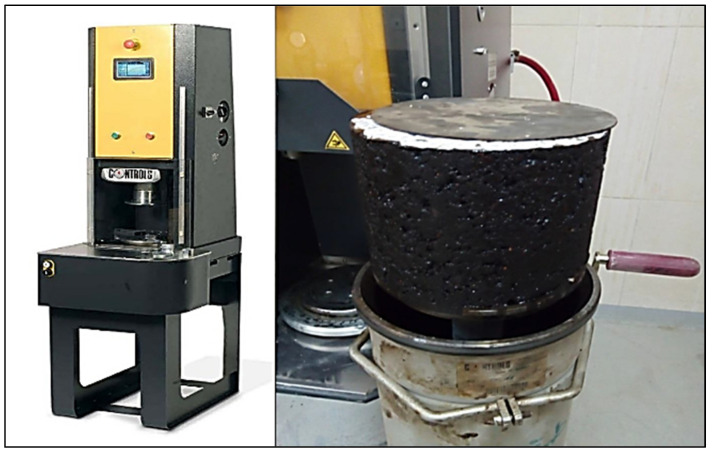
Asphalt sample compacted using SGC.

All of the produced specimens are compacted using the Superpave gyratory compactor (SGC), as shown in Figure 4, in line with AASHTO T 312, 2015 [36] and the Superpave mix design procedure (NCHRP-752, 2013) [37]. 

The common compaction method is set on three different levels of gyrations (N_design_, N_initial_, and N_max_), where N refers to the number of gyrations used to determine the maximum theoretical density of the prepared specimens. Medium to high traffic levels were used to select the N_design_ at 100 gyrations from NCHRP-714, 2012 [38]. The level of compaction sets from SHRP-A-407 [39] suits Babylon’s city climate and traffic in Iraq, as shown in Table 13.

After selecting the aggregate design structure, a trial mixture was conducted to estimate the binder content based on the Asphalt Institute Manual, 2014. Normally, a trial binder content was used depending on the nominal maximum size of the aggregate, which is 12.5 mm for the surface layer. In our case, the elected content equals 5%, depending on the design procedure of the Asphalt Institute. This procedure aims to calculate the amount of binder required to achieve 4% air content in the compacted mixes. In order to conduct the maximum theoretical and bulk-specific gravity (Gmm and Gmb) in line with AASHTO T 209 [40] and AASHTO T 166 [41], respectively, Superpave specimens were compacted using 4790 gm at the chosen binder content for trial. At 5% binder, the trial mixture’s air content deviated from 4% or 96% Gmm. Therefore, after multiple trials, the target has reached an estimated 4.9% binder content, which corresponds to the desired air content. All related design equations for predicting binder content were applied following AASHTO R 35 [42]. The volumetric properties of trial mixtures at estimated binder content are presented in Table 14.

As a result of compacting four different types of mixtures, each has a certain amount of binder; as listed below, the optimum binder content is identified. Three asphalt specimens have been compacted for each binder percent.

- The first trial mixture is mixed at the estimated content of the binder;
- The second trial mixture is mixed at the estimated content of the binder minus 0.5%;
- The third trial mixture is mixed at the estimated content of binder plus 0.5%;
- The fourth trial mixture is mixed at the estimated content of binder plus 1.0%.

The volumetric properties of the developed mixtures are obtained by considering air voids, VFA, and VMA and the value of Gmm at each gyration level (NCHRP-673, 2011) [44]. The optimum binder content is fixed at 4% air content, consistent with AASHTO R 35.

AASHTO M 323 [43] is used as the basis for handling the RAP in the HMA mixtures. Choosing the proper grade of virgin binder depends on the viscosity and penetration of the recovered RAP binder; hence, special blending charts are needed only for mixes having a high percentage of RAP, more than 25%.

The combined blend between the fresh binder and the aged RAP binder is dense, with high viscosity, and is made denser by increasing the percentage of RAP within the mixture. Therefore, a radical solution is needed by providing a softer virgin binder or adopting recycling agents/rejuvenators to mix with the recycled mixtures. Recovering basic characteristics of the aged binder within the old pavement is implemented using different viscous oils. The whole process can be summarized by adding new maltenes to dilute the asphaltenes that have formed due to time. 

The amount of virgin binder that can be added to the recycled HMA mixture depends on the amount of RAP in the mix; therefore, the Asphalt Institute and AASHTO M 323 developed an equation to calculate the added percentage of fresh binder [45].

(1) $$P_{R}=P_{C}-\left(P_{A}\times P_{P}\right)$$

where *P_R_* is the percentage of virgin binder in the mix, *P_A_* is the percentage of RAP binder, *P_C_* is the total binder content in the mix, and *P_P_* is the percentage of RAP in the mix.

### 2.3. Rolling Thin Film Oven Test Aging (RTFO)

**Figure 5:**
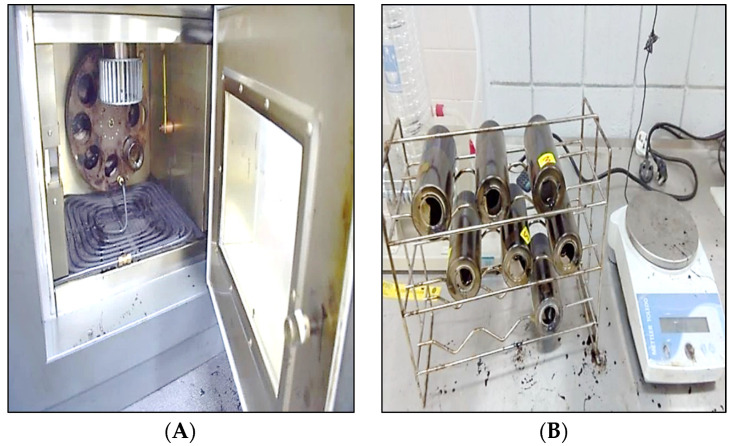
RTFOT apparatus. (**A**) Rotating oven; (**B**) RTFO bottle.

The RTFO test is performed to change the binders’ condition from unaged to short-term aged by following the AASHTO T 240 test method [46]. The apparatus and an RTFO bottle are shown in Figure 5. It stimulates the binder’s aging during manufacturing and placement in the field. The RTFO chamber was preheated at 163 °C. Subsequently, the sample bottles, after cooling down, were carefully put into a carousel oven. The carousel rotates at a constant speed of 15 RPM for 85 min. Throughout the rotation of the carousel, the temperature of the oven and the airflow rate into bottles were maintained at 163 °C and 4 lit/min, respectively. The RTFO residue was tested within 72 h.

### 2.4. Measuring the Viscosity

**Figure 6:**
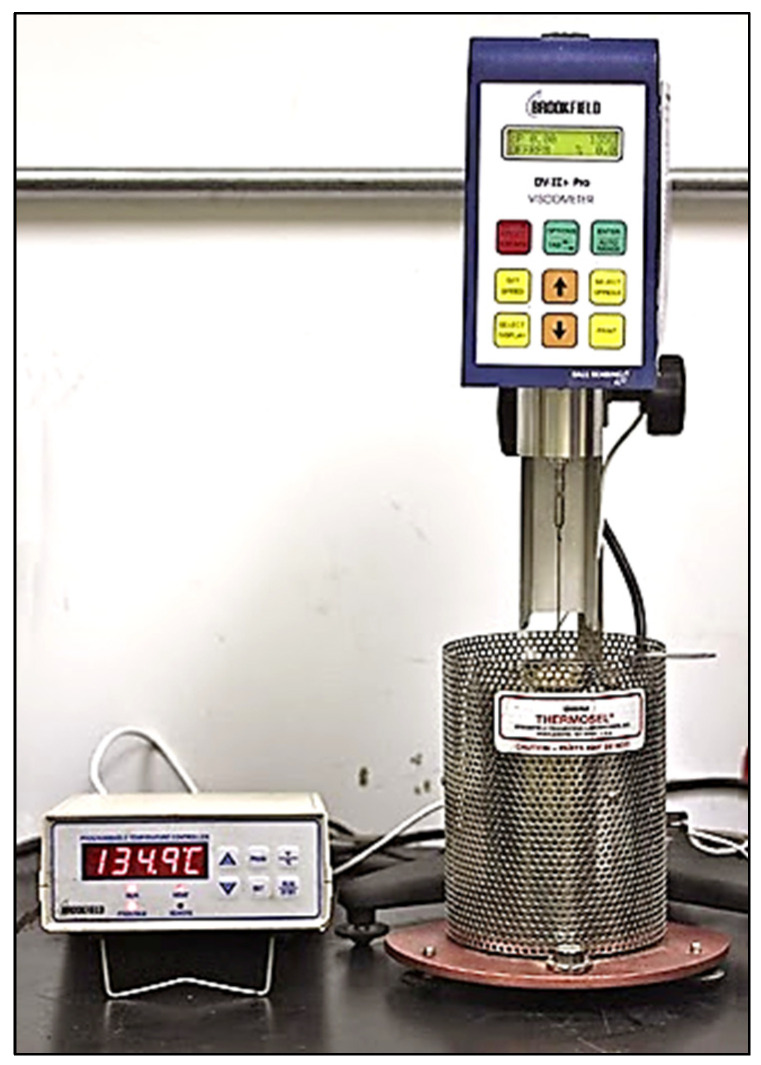
Rotational Brookfield viscometer.

**Figure 7:**
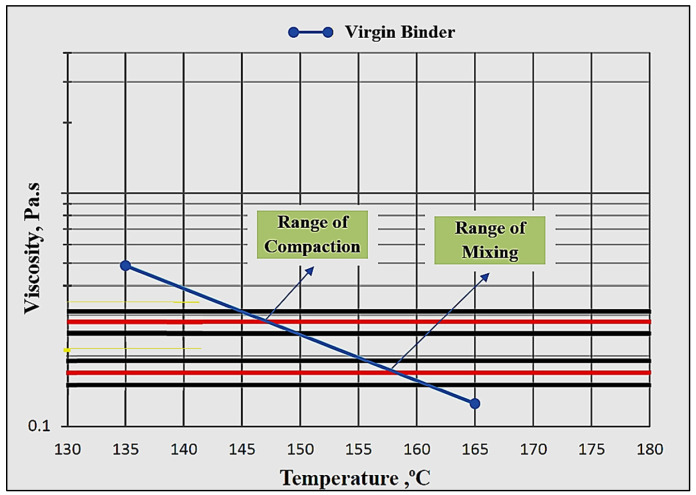
Viscosity–temperature logarithmic chart of virgin binder.

Viscosity for the modified and unmodified asphalt binders is usually determined by using a rotational Brookfield viscometer, as shown in Figure 6, through the traditional standard method ((ASTM D4402) [47]/(AASHTO T316) [48]). The rotational Brookfield (RV) test is performed at constant rotational speed (20 RPM) of a cylindrical spindle while submerged in a binder. The amount of binder used varies with the spindle size, at most 11 gm. Viscosity values were obtained for the unmodified binders at pumping and handling temperatures to prepare the logarithm of viscosity versus the logarithm of temperature charts for the unmodified binder, as shown in Figure 7, that can be used to determine equiviscous mixing and compaction temperatures corresponding to viscosity ranges of 0.17 ± 0.02 Pa.s and 0.28 ± 0.03 Pa.s, respectively. Consequently, the viscosity values were measured at 135 °C and 165 °C, then a straight line was drawn to connect these two points.

Several trials of the modified combined blend (the recovered aged binder of RAP + rejuvenators) were conducted at each percent of RAP within the HMA mixtures using the rotational Brookfield viscometer. The aim of the trials is to determine the potential effect of the rejuvenators by achieving a considerable level of viscosity similar to or close enough to the level of the control virgin binder. The adopted percentages of the recycling agents are illustrated in Table 15.

### 2.5. Dynamic Shear Rheometer

**Figure 8:**
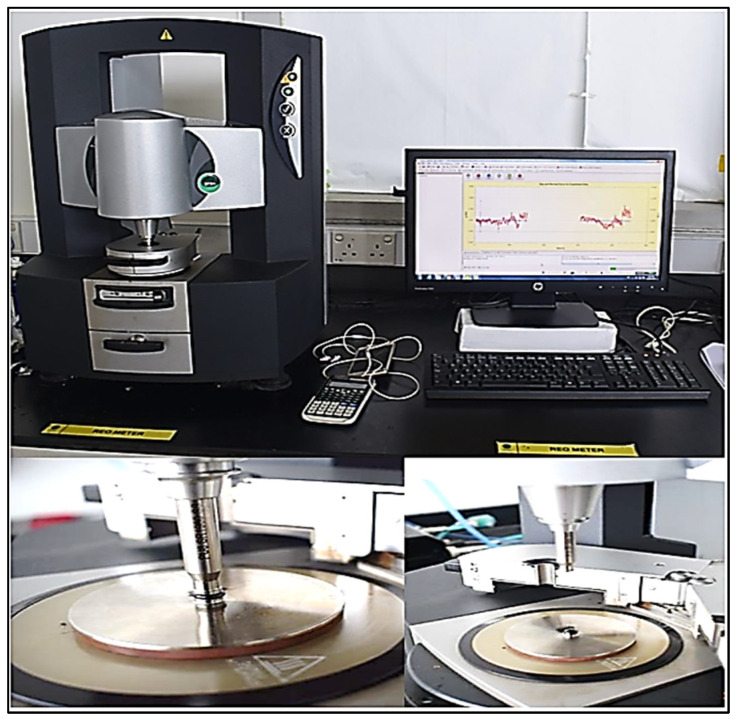
Kinexus DSR.

A DSR test was performed to evaluate the effect of different types of rejuvenators within the recycled HMA on the rheological properties by determining the complex modulus and phase angle at high temperatures under torques ranging from 0.5 μN·m to 200 μN·m according to AASHTO T315 [49]. The DSR works by converting applied torque to strain while the sample is saved in an active hood (thermally controlled test chamber) throughout testing to accurately and dependably manage the temperature within ±0.1 °C tolerance. When testing unaged and RTFO binders at high temperatures, the (G*/sin δ) value is employed as the rutting index. According to a general rule, a larger (G*/sin δ) indicates a high permanent deformation confrontation. Kinexus DSR from Malvern Instruments was utilized in this test, as shown in Figure 8.

### 2.6. Hamburg Wheel-Track Test

**Figure 9:**
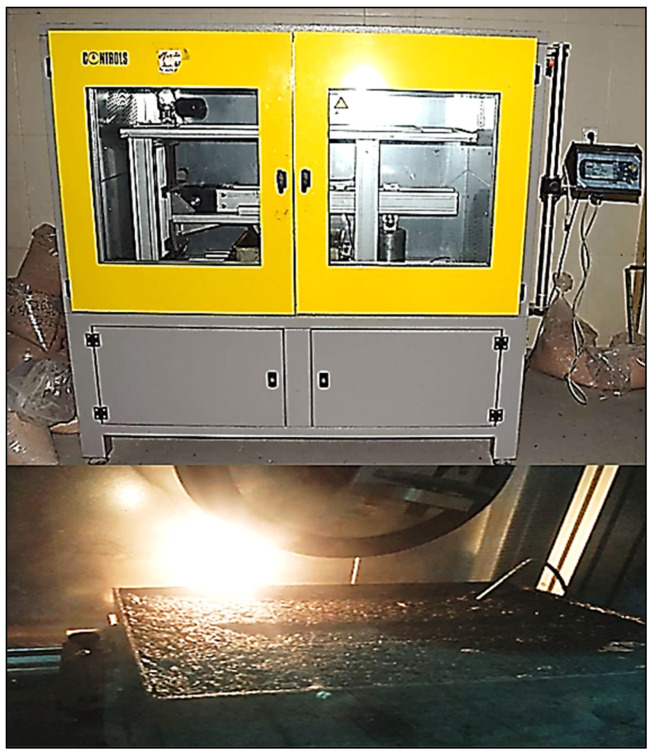
Operating WTT.

In line with AASHTO T-324 [50], the wheel tracking test (WTT) was applied to simulate the rutting susceptibility of laboratory-prepared asphalt mixtures to deform under load, as shown in Figure 9. The permanent deformations (rut depth) produced by repeated wheel load cycles at a certain temperature were simulated using the WTT. A rubber wheel that is 203.2 mm in diameter and 47 mm wide can be moved across a test specimen using the mechanical wheel tracking mechanism. The table of the device has an alternate harmonic spanning a distance of 230 ± 5 mm at 52 RPM with the wheel in one forward and backward motion (two passes), which is considered one load cycle. 

The wheel has a solid rubber tire with a diameter of 200 mm and a maximum speed of roughly 0.305 m/s. The standard wheel load is 700 ± 10 N while the wheel tracker is located in temperature-controlled cabinets up to 65 ± 1.0 °C and is designed to capture at least two readings at 25 places along a 10 cm line at the center of the sample immediately along the wheel route. The WTT temperature was set at 60°C for this study to represent the most extreme high-performance temperature in hot locations. The WTT test is completed when the wheel has completed 10,000 revolutions over the specimen or when the rut depth exceeds 20 mm.

#### Preparation Process of the Samples

**Figure 10:**
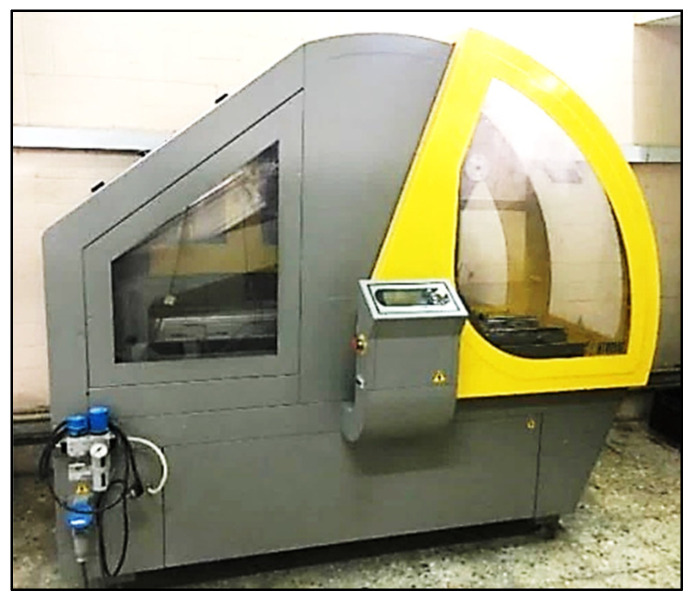
Pneumatic compactor.

**Figure 11:**
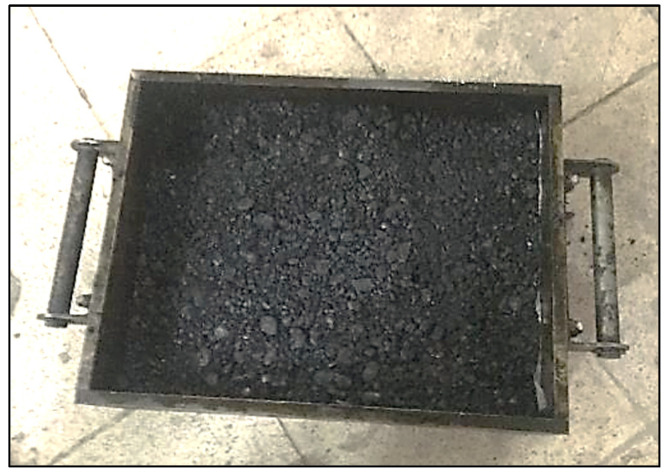
Sample rectangular mold.

A pneumatic roller compactor, as shown in Figure 10, was used to compact a slab sample in a rectangular mold, as shown in Figure 11, of 400 mm × 300 mm × 120 mm. With a batch of 11 kg, two slab samples were implemented for each type of the adopted mixture to generate samples with a thickness of 40 mm (thickness must be at least twice the nominal aggregate maximum size) (EN 12697-22) [51]. Before applying the test, each compacted slab’s air void was examined. If the air void exceeded 4% ± 0.5%, the compacted slab was disposed of and replaced. AASHTO R 30 [52] required that HMA samples be conditioned for 4 h at 135 °C. 

## 3. Results and Discussion

**Figure 12:**
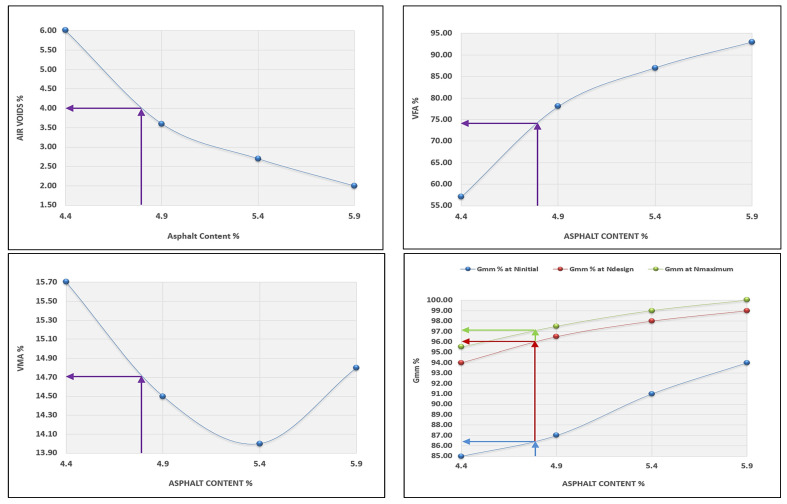
Volumetric properties of trial mixtures.

The optimum binder content for the designed HMA mixtures is predicted at 4% air content. The rest of the volumetric properties—VFA, VMA, and the level of Gmm at N_max_, N_design_, and N_initial_—have been figured out in line with NCHRP-673, 2011 [53], as shown in Table 16 and Figure 12.

The optimum content of the binder is 4.8%, determined at 4% air voids in line with AASHTO M 323 for Superpave mix design. The volumetric properties of the generated specimens at the optimum binder content are illustrated in Table 17.

The added percentage of fresh or virgin binder to the recycled HMA mixtures at each percent of RAP is tabulated in Table 18.

### 3.1. Blending Process of the Rejuvenators and the Virgin Binder

**Figure 13:**
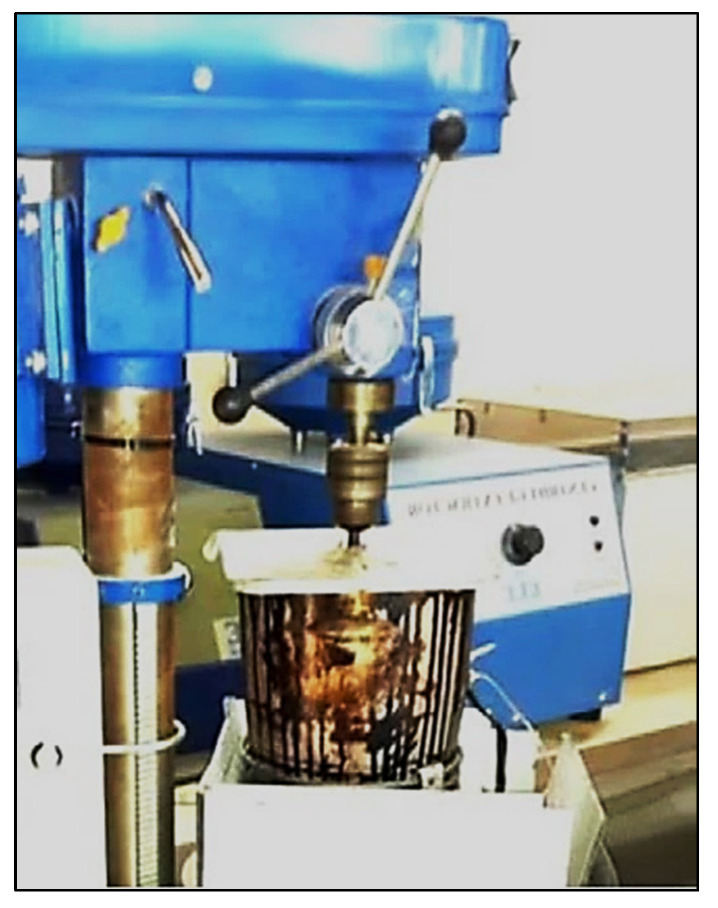
Blending process.

The added quantities of WCO, WEO, and DSO are calculated by the weight of the aged RAP binder in the recycled mix, whereas it depends mainly on the percentage of RAP within the mix. In contrast, the added dosage of SonneWarmix RT^TM^ is calculated by the weight of the total binder in the mix. The mixing process is a wet process in which the rejuvenators are added to the asphalt binder before introducing it into the asphalt concrete mixture. All the used rejuvenators are added to the asphalt binder in a blending machine at a blending speed of 600 RPM for half an hour at 160 °C [53], as shown in Figure 13.

### 3.2. Measuring the Variation in Viscosity of the Aged Blend of Binders Due to Adding the Used Rejuvenators

**Figure 14:**
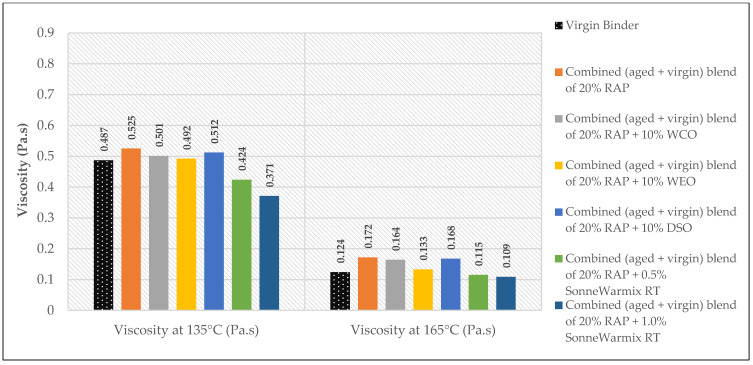
Rotational viscosity of combined (aged + virgin) binder blend of 20% RAP.

**Figure 15:**
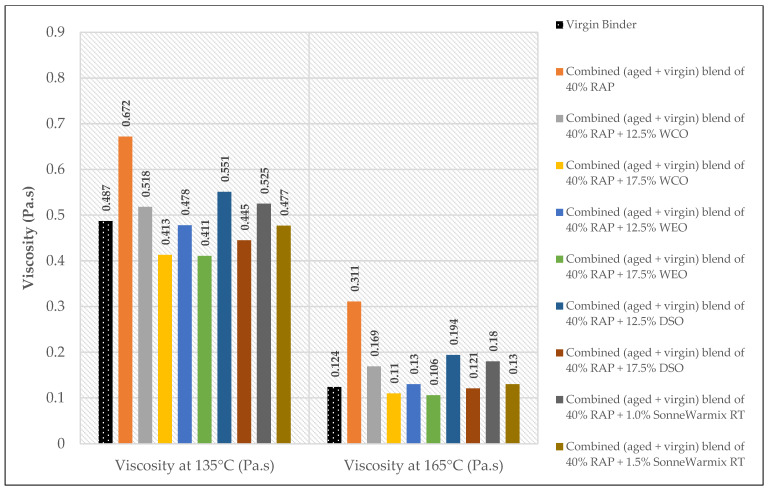
Rotational viscosity of combined (aged + virgin) binder blend of 40% RAP.

**Figure 16:**
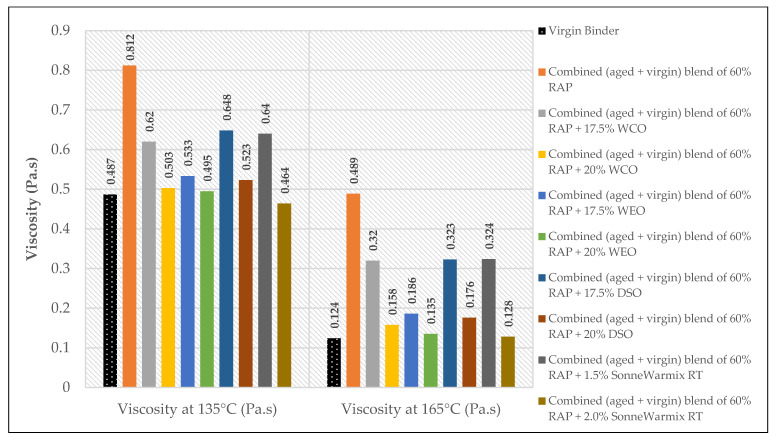
Rotational viscosity of combined (aged + virgin) binder blend of 60% RAP.

The effects of the rejuvenators on the viscosity levels of the combined blend of binders (aged + virgin) are demonstrated in Figure 14, Figure 15 and Figure 16.

Depending on the added percentages of RAP within the recycled mixtures, the effects of the rejuvenators on the viscosity behavior are presented.

#### 3.2.1. Combined Blend of (Aged + Virgin) Binder + WCO

**Figure 17:**
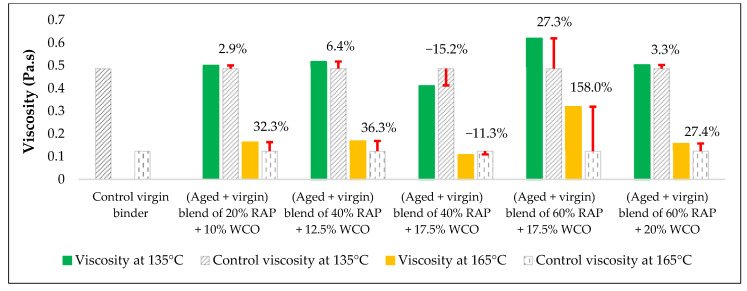
The change in viscosity level from adding WCO as a rejuvenator to the combined blend of binders in comparison to the control viscosity of the virgin binder.

Adding WCO to the aged blend of binders affected the viscosity of the blend, as demonstrated in Figure 17. WCO tries to push the aged blend to regain the physical characteristics that were lost due to aging by enhancing its molecular structures and makes it similar in performance to the virgin binder. In other words, WCO has cured the hard and stiff aging defects in the aged blend of binders. By comparing this result with the findings of Dokandari et al. [54], a similarity in the methodology is noticed, but the current study offers new parameters and evaluation tests to continue the prior study. Both percentages of RAP-binder and WCO within the blend significantly affect the viscosity values; therefore, at high percentage of RAP (>25%), two percentages of WCO have been mixed with each percent of RAP in order to reach the optimum content of the used rejuvenator that could rejuvenate the aged blend effectively. 

#### 3.2.2. Combined Blend of (Aged + Virgin) Binder + WEO

**Figure 18:**
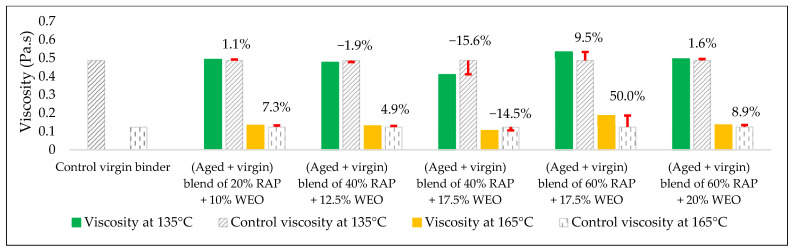
The change in viscosity level from adding WEO as a rejuvenator to the combined blend of binders in comparison to the control viscosity of the virgin binder.

Adding WEO to the aged blend of binders affected the viscosity of the blend, as demonstrated in Figure 18. The unique structure of WEO works to enhance the structures of the aged binder at a molecular level with sufficient aromatic content, which results in cohesive bonding through modifying the aged binder components and rejuvenating the lost physical and chemical characteristics. This result is supported by the findings of Woszuk et al. [55], in addition to adding new boundary conditions in terms of mixing and compaction temperatures to develop the previous work. The optimal content of the utilized rejuvenator that could revive the aged blend, especially at high RAP content (>25%), was determined by mixing two percentages of WEO with each percent of RAP.

#### 3.2.3. Combined Blend of (Aged + Virgin) Binder + DSO

**Figure 19:**
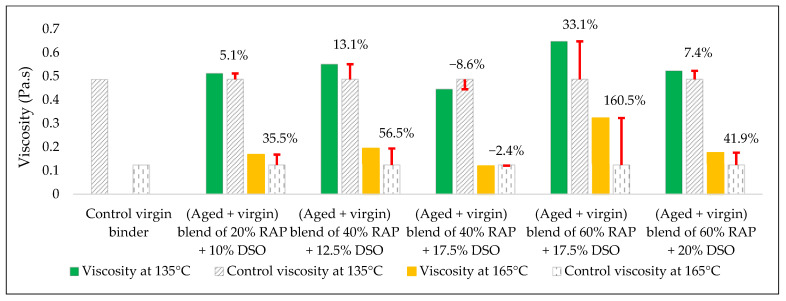
The change in viscosity level from adding DSO as a rejuvenator to the combined blend of binders in comparison to the control viscosity of the virgin binder.

Adding DSO to the aged blend of binders affected the viscosity of the blend, as demonstrated in Figure 19. DSO consists of fatty acids, which are long aliphatic chains of carboxylic acids with the ability to restore the original properties of the aged binder as a rejuvenator. This result ties well with the results gained by Mirhosseini AF. et al. [19]. In addition, the current study utilized several DSO proportions that were mixed with each percent of RAP in order to conduct the optimal rejuvenator content as a step of development.

#### 3.2.4. Combined Blend of (Aged + Virgin) Binder + SonneWarmix RT^TM^

**Figure 20:**
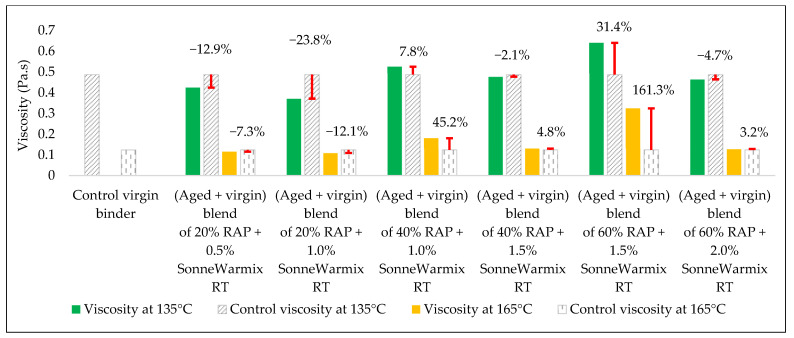
The change in viscosity level from adding SonneWarmix RT^TM^ as a rejuvenator to the combined blend of binders in comparison to the control viscosity of the virgin binder.

Adding SonneWarmix RT^TM^ to the aged blend of binders affected the viscosity of the blend, as demonstrated in Figure 20. SonneWarmix RT^TM^ reduces the damage caused by oxidative aging of binder and tries to regain the lost properties. Overall, the current findings are in accordance with findings reported by Zinke S. et al. [29], wherein new performance tests have been implemented to continue the previous work.

In terms of viscosity enhancement of aged binders compared to the control, at 20% RAP, the performance of noncommercial rejuvenators was better than the commercial ones for all the adopted percentages at 135 °C, whereas at 165 °C, best results were achieved using either 10% WEO or 0.5% SonneWarmix RT^TM^, which have the same effect on the viscosity level. 

At 40% RAP, using 12.5% and 1.0% of the noncommercial and commercial rejuvenators, respectively, the viscosity level was modified better using the noncommercial rejuvenators at both 135 °C and 165 °C, in comparison to the control binder. Increasing the rejuvenator percentages to 17.5% and 1.5% at 40% RAP for both types, respectively, led to effectively improving the viscosity of the aged binder by the commercial rejuvenator at 135 °C, whereas at 165 °C, the best result was achieved using the DSO rejuvenator in comparison to the control binder. 

Furthermore, at 60% RAP, using 17.5% and 1.5% of the noncommercial and commercial rejuvenators, respectively, the viscosity level was modified better using the noncommercial rejuvenators at both 135 °C and 165 °C in comparison to the control binder. In addition, increasing the rejuvenator percentages to 20% and 2.0% at 60% RAP for both types, respectively, led to the best result using the WEO rejuvenator at 135 °C, whereas at 165 °C, the best result was achieved using the commercial rejuvenator in comparison to the control binder.

**Figure 21:**
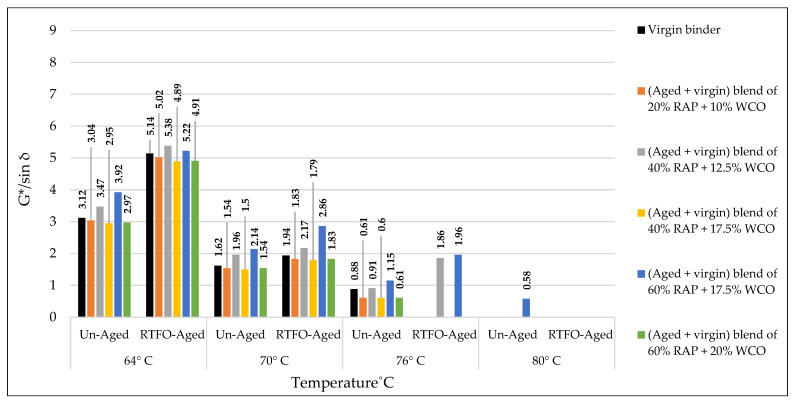
Rutting index (G*/sin δ) for the aged blends of binders rejuvenated by WCO.

**Figure 22:**
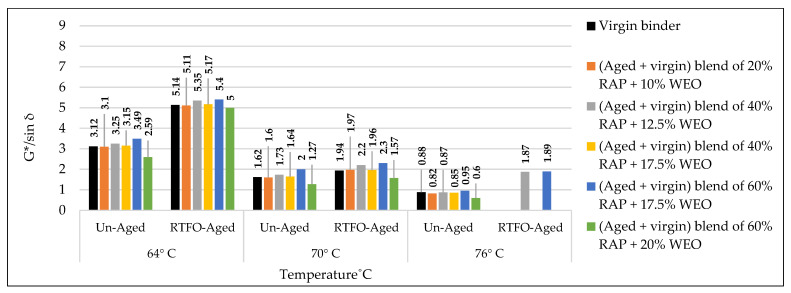
Rutting index (G*/sin δ) for the aged blends of binders rejuvenated by WEO.

Increasing and decreasing the levels of viscosity of the combined blend of binders after mixing with the rejuvenators, as presented in Figure 19, Figure 20, Figure 21 and Figure 22, can be interpreted depending on the percentage of the aged binder within the blend. As high as the percentage of aged binder, a high percentage of the rejuvenator is required to equalize the stiffness of the blend, and vice versa. 

Therefore, two individual percentages of the used rejuvenators were used at each percent of RAP, as previously shown in Table 15. In addition, it also depends on the characteristics of the rejuvenators used. This result is supported by the findings of Zaumanis et al. [56], wherein new mixing parameters are presented in the current research to continue the prior study.

### 3.3. Rutting Potential Indicator (G*/sin δ) for Binders

According to AASHTO T315, the DSR test was implemented to predict the effect of the used rejuvenators on the rheological characteristics of the combined blend of binders, as shown in Table 19 and Table 20. The effects of rejuvenators on the rutting index (G*/sin δ) can be explained due to changing the viscosity of the combined blends of binders, whereas the rejuvenators work to enhance the characteristics of the aged binder and minimize its hard viscosity. In other words, the rejuvenators change the performance grade (PG) of the aged blend and make it similar to the virgin one. A similar pattern of results was obtained previously by Shen et al. [47], except the current study offers a variety in the used rejuvenators under different circumstances.

The effects of adding recycling agents/rejuvenators on the levels of rutting index (G*/sin δ) for the aged blend of binders are demonstrated below for the unaged and aged samples of binders.

**Figure 23:**
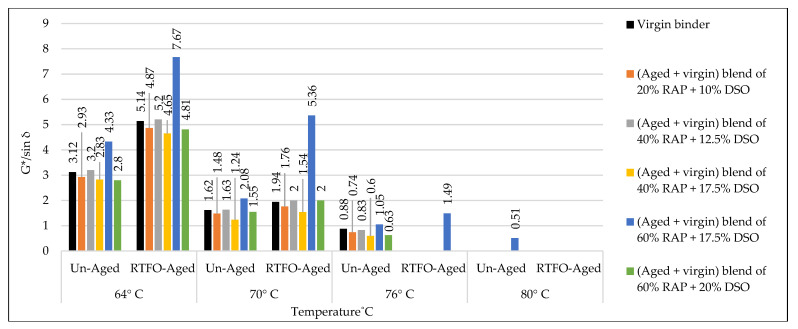
Rutting index (G*/sin δ) for the aged blends of binders rejuvenated by DSO.

**Figure 24:**
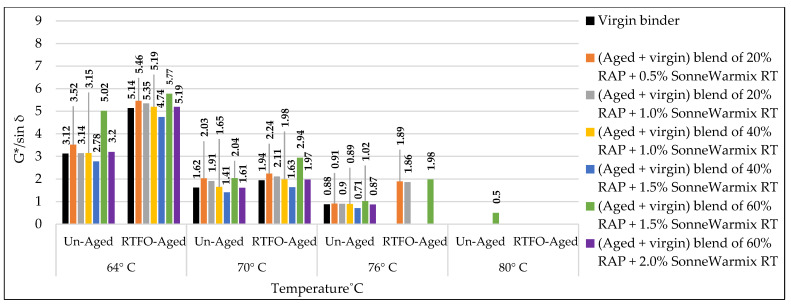
Rutting index (G*/sin δ) for the aged blends of binders rejuvenated by SonneWarmix RT^TM^.

1. Using WCO within the aged blend of binders has proven to be very efficient in stabilizing the rutting index (G*/sin δ) equally with the control virgin binder, as shown in Figure 21. Best results were achieved by the rejuvenated blends of 40% RAP + 12.5% WCO and 60% RAP + 17.5% WCO, whereas the levels of G*/sin δ for the noted blends of binders are relatively high in comparison to the virgin ones, which indicates high resistance to rutting deformation. WCO has motivated the aged blend to restore the physical qualities lost due to aging by improving its molecular structure and making it similar to the virgin binder. The current results are directly in line with prior findings of Dokandari et al. [54]. In addition, it adds new testing parameters to the previous ones.
2. Using WEO within the aged blend of binders has proven to be very reliable in stabilizing the rutting index (G*/sin δ) equally with the control virgin binder, as shown in Figure 22. Best results were achieved by the rejuvenated blends of 20% RAP + 10% WEO, 40% RAP + 12.5% WEO, 40% RAP + 17.5% WEO, and 60% RAP + 17.5% WEO, whereas levels of G*/sin δ for the noted blends of binders are higher or similar to the level of virgin binder, which indicates high resistance to rutting deformation. Through the modification of the old binder components and restoration of the lost physical and chemical properties, the distinctive WEO structure works to improve the aged binder’s molecular structures and provide cohesive bonding. Woszuk et al. [55] support these outcomes using the same testing procedure at only two degrees of test temperatures.
3. Using DSO within the aged blend of binders has proven to be very effective in stabilizing the rutting index (G*/sin δ) equally with the control virgin binder, as shown in Figure 23. A high level of rutting index was achieved by the rejuvenated blends of 40% RAP + 12.5% DSO and 60% RAP + 17.5% DSO, which indicates a high resistance to rutting deformation. Fatty acids, which are long aliphatic chains of carboxylic acids and are part of DSO composition, have the power to revive the aged binder in terms of physical and chemical properties. These results are consistent with the findings of Mirhosseini AF. et al. [19].
4. Using SonneWarmix RT^TM^ within the aged blend of binders has proven to be very efficient in stabilizing the rutting index (G*/sin δ) equally with the control virgin binder, as shown in Figure 24. A high level of rutting index was achieved by almost all of the developed binder blends similar to or higher than the level of control binder, except the blend of 40% RAP + 1.5% SonneWarmix RT^TM^, which has a relatively low value of rutting index. SonneWarmix RT^TM^ mitigates binder oxidative aging damage and attempts to restore lost characteristics. The current results are consistent with those reported by Zinke S. et al. [29], although they used different percentages of SonneWarmix RT^TM^ and a different testing approach.

Regarding rutting index (G*/sin) modification of the unaged and RTFO-aged binders, the performance of the commercial rejuvenator at 20% RAP was better than noncommercial ones at all the adopted percentages under 64 °C, 70 °C, and 76 °C test temperatures compared to the control level. At 40% RAP, the performance of the noncommercial rejuvenator was better than the commercial ones at all the adopted percentages under 64 °C, 70 °C, and 76 °C test temperatures in comparison to the control level. At 60% RAP, the performance of the commercial rejuvenator at 20% RAP was better than noncommercial ones at all the adopted percentages under 64 °C, 70 °C, and 76 °C test temperatures compared to the control level.

The effect of the rejuvenators on the rutting index (G*/sin δ) of the aged binder can be explained by enhancing the aged characteristics of the binder and changing its PG to be similar to the virgin one or even better, leading to generating recycled mixtures with significant resistance to rutting stresses. 

In addition, each type of the used rejuvenators has its effect in enhancing the rutting index (G*/sin δ) depending on its characteristics and the recommended mixing dosage. This result ties well with the prior study implemented by Al-Saffar et al. [19].

### 3.4. The Results of WTT

All the related results for the Hamburg test are demonstrated in Table 21, considering all the effects of the variation in binder grade at each type and percentage of the used rejuvenators.

**Figure 25:**
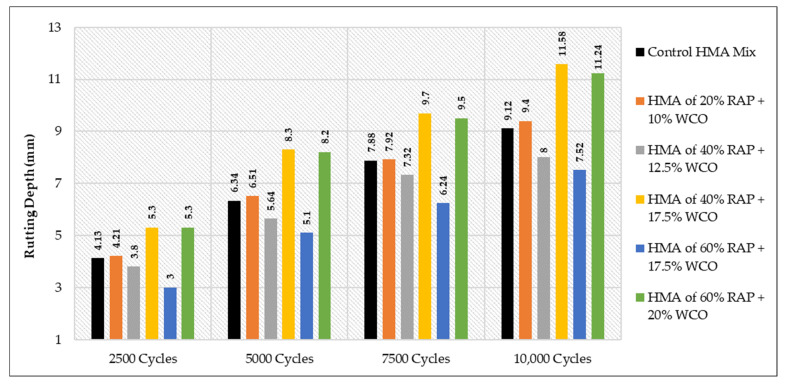
Rutting depths of recycled HMA mixtures rejuvenated by WCO.

**Figure 26:**
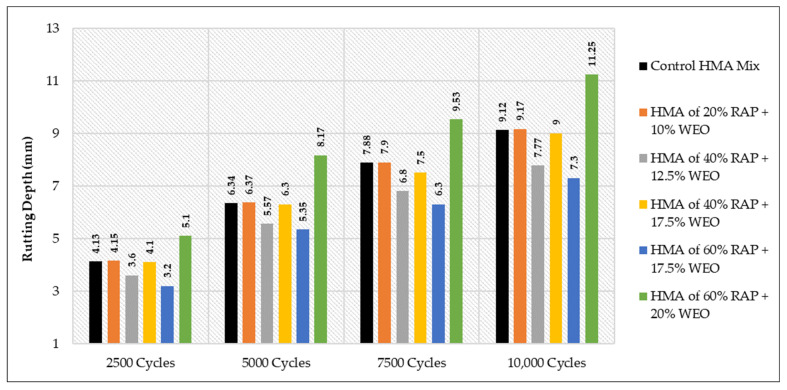
Rutting depths of recycled HMA mixtures rejuvenated by WEO.

**Figure 27:**
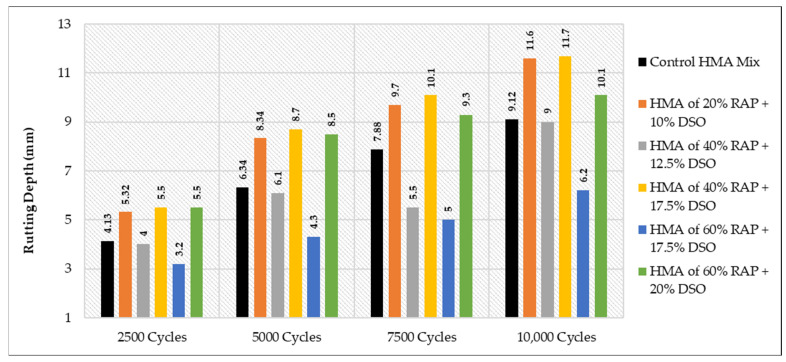
Rutting depths of recycled HMA mixtures rejuvenated by DSO.

**Figure 28:**
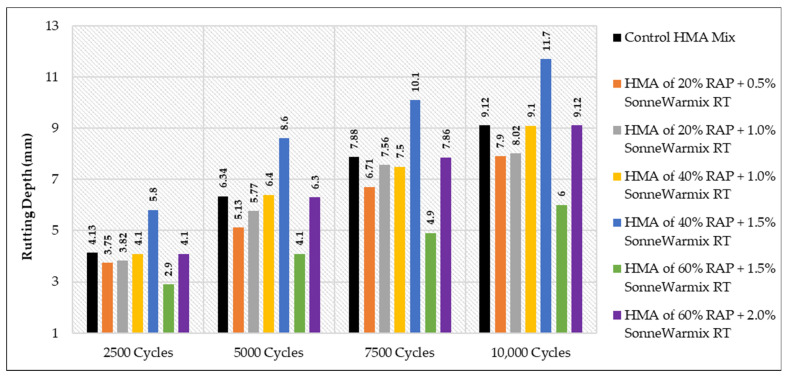
Rutting depths of recycled HMA mixtures rejuvenated by SonneWarmix RT^TM^.

It is worth noting that the obtained rut depth for 10,000 cycles was 11.7 mm when the 40% of HMA RAP +1.5 SonneWarmix RT^TM^, whereas the resulting depth of 60% of HMA RAP +1.5 SonneWarmix RT^TM^ was noted as 7.6 mm. This can be explained due to the rigidity properties of the 60% mixture that behave in this manner under rutting, in comparison with the control mixture. The most reliable interpretation for the results of WTT is listed below for each case:

- As shown in Figure 25, HMA mixtures of RAP + WCO showed noticeable levels of rutting resistance in comparison to the control mixture. The mixtures that accomplished the best performance under rutting were 40% RAP + 12.5% WCO and 60% RAP + 17.5% WCO.
- As shown in Figure 26, HMA mixtures of RAP + WEO showed considerable levels of rutting resistance in comparison to the control mixture. Mixtures that accomplished the best performance under rutting were 20% RAP + 10% WEO, 40% RAP + 12.5% WEO, 40% RAP + 17.5% WEO, and 60% RAP + 17.5% WEO.
- As shown in Figure 27, HMA mixtures of RAP + DSO showed great levels of rutting resistance in comparison to the control mixture. Moreover, mixtures that accomplished the best performance under rutting were 40% RAP + 12.5% DSO and 60% RAP + 17.5% DSO.
- As shown in Figure 28, HMA mixtures of RAP + SonneWarmix RT^TM^ showed remarkable levels of rutting resistance in comparison to the control mixtures. All the rejuvenated mixtures using SonneWarmix RT^TM^ accomplished the best performance under rutting, except the HMA mixture of 40% RAP + 1.5% SonneWarmix RT^TM^, which showed a low level of rutting resistance in comparison to the control mixture.

Overall, the performance of rejuvenated HMA mixtures under rutting was implemented by using both rejuvenator types. At 20% RAP, the performance of the commercial rejuvenator at all the conducted percentages proved to be very effective in reducing the rutting depth in comparison to the control mix under all test cycles. At 40% RAP, the performance of noncommercial rejuvenators at all the conducted percentages proved to be very effective in reducing the rutting depth in comparison to the control mix under all test cycles. At 60% RAP, the performance of the commercial rejuvenator at all the conducted percentages proved to be very effective in reducing the rutting depth in comparison to the control mix under all test cycles (Table 22). The optimum percentages of the used commercial and noncommercial rejuvenators within the recycled HMA mixtures were determined in terms of equalizing the aged characteristics of the combined blends of binders and enhancing the rutting resistance, as shown in Table 22.

## 4. Conclusions

In this research, three noncommercial rejuvenators (i.e., WCO, WEO, and DSO) and one commercial rejuvenator in asphaltic mixtures incorporating RAP at three levels (20%, 40%, and 60%), were investigated. All the asphaltic mixtures were prepared and evaluated under the Superpave design method. The elected aggregate gradation for preparing the mixtures was for surface course mixed with 20%, 40%, and 60% RAP. The conclusions of this research are summarized as follows:

- The optimum percentages of the used noncommercial rejuvenators (i.e., 20% RAP) were within the recycled HMA mixtures. whereas for the commercial rejuvenator (i.e., 0.5–1.0% SonneWarmix RT^TM^) it was determined by the total weight of binder content.
- All the obtained results of the rutting tests of the rejuvenated recycled mixtures were confirmed by the achieved results using rutting index (G*/sin δ) with respect to the rejuvenated blends of binders.
- The best resistance to rutting stresses was accomplished at 20 and 60% RAP by the rejuvenated recycled HMA mixtures using SonneWarmix RT^TM^, whereas the best rutting resistance at 40% RAP was accomplished by the rejuvenated recycled mixtures using all three types of noncommercial additives (i.e., WCO, WEO, and DSO) in comparison to the control mixture.
- All the rejuvenated binders proved to be stiff and elastic with the ability to regain their shapes after removing the deformation. This research accomplished that by using the optimal dosages of commercial and noncommercial rejuvenators.
- All the used rejuvenators performed very well in minimizing the high viscosity of the combined aged blends of binders to acceptable levels, in comparison to the control viscosity of virgin binders.
- The working mechanism of the adopted rejuvenators involved changing the performance grade (PG) of the aged binder to a softer grade similar to that of the control virgin binder.

The current study contributes to the previous findings of researchers by conducting a comprehensive investigation using two types of recycling agents with affordable prices to rejuvenate recycled HMA mixtures and trying to compare the performance of each type of rejuvenator in terms of rutting resistance. Despite that, one of the limitations of this study is the lack of some tests for the used asphalt binder, including binder pressure aging vessel (PAV) and frequency sweep test (FST). Thus, it is recommended for future work to implement an extensive investigation regarding the aforementioned tests to overcome this limitation.

## Figures and Tables

**Table 1:** Physical characteristics of RAP.

Property	Bulk s.g. of Coarse RAP	Bulk s.g. of Fine RAP	Absolute Viscosity@60 °C (Poise)	Binder Content	Moisture Content
Result	2.512	2.501	18,900	3.6%	1.03%
Standards	ASTM C-127/C-128		ASTM D2171	AASHTO T 308	AASHTO T 255

**Table 2:** RAP’s gradation.

English Sieves	Standard Sieves (mm)	RAP Gradation (% Passing)
3/4”	19	100
1/2”	12.5	92.4
3/8”	9.5	81.3
No.4	4.75	49.5
No.8	2.36	42.6
No.50	0.3	9.5
No.200	0.075	1.9

**Table 3:** Aggregate gradation of a surface layer.

English Sieves	Standard Sieves (mm)	Specification Limit	Surface Layer Gradation
3/4”	19	100	100
1/2”	12.5	100-90	96
3/8”	9.5	90-76	82
No.4	4.75	74-44	60
No.8	2.36	58-28	44
No.50	0.3	21-5	14
No.200	0.075	10-4	7

**Table 4:** Physical characteristics of the aggregate.

Property	Bulk s.g.	Apparent s.g.	Los Angeles (Abrasion)	Angularity	Water Absorption
Coarse aggregate	2.532	2.661	22%	94%	1.0%
Fine aggregate	2.583	2.693	/	/	0.74%
ASTM Standards	C-127/C-128		C-131	D-5821	C-127/C-128

**Table 5:** Physical characteristics of the used binder.

Property	Penetration@25 °C, 100 gm, 5 s	Ductility@25 °C, 5 cm/min	Kinematic Viscosity@135 °C	Flashpoint (Cleveland open cup)	Softening Point	s.g.@25 °C
Result	45	133	384 cSt	241 °C	54 °C	1.05
ASTM Standards	D5	D113-99	D-2170	D92	D36	D70

**Table 6:** Physical characteristics of the Portland cement as filler.

Property	Fineness, Blaine, (cm^2^/gm)	s.g@25 °C	Passing Sieve No.200
Result	3210	3.15	97%

**Table 7:** Physical characteristics of WCO.

Property	s.g.@25 °C	Kinematic Viscosity@40 °C	Dynamic Viscosity mPa.s	Flash Point
Result	0.942	46.31	42.45	306 °C
ASTM Standards	D1298	D445	D7042	D93

**Table 8:** Chemical characteristics of WCO.

Type of Fatty Acid and Formulation	Percentage of the Weight
Linoleic acid/C18:2 (Cis)	25%
Lignoceric acid/C24:0	0.08%
Pentadecylic acid/C15:0	0.04%
Margaric acid/C17:0	0.05%
Myristic acid/C14:0	0.63%
Lauric acid/C12:0	0.12%
Vaccenic acid/C18:1 t	0.3%
α-Linolenic acid/C18:3 alpha	0.8%
Palmitoleic acid/C16:1	0.3%
Palmitic acid/C16:0	28.4%
Oleic acid/C18:1 (Cis 9)	39.8%
Stearic acid/C18:0	4.2%
Linoleic acid/C18:2 t	0.17%

**Table 9:** Physical characteristics of WEO.

Property	Color	s.g.@25 °C	Absolute viscosity@60 °C (poise)	State
Result	Black to dark brown	0.877	0.56	Oily
ASTM Standards	/	D1298	D7042	/

**Table 10:** Physical characteristics of DSO.

Property	Color	Odor	Absolute Viscosity@60 °C (poise)	s.g @ 25 °C	Moisture Content	Texture
Result	Yellow or pale yellow	pleasant	0.17	0.92	9.6%	viscous
ASTM Standards	/	/	D7042	D1298	D6304	/

**Table 11:** Chemical characteristics of DSO.

Fatty Acid Type	Lauric	Myristic	Linoleic	Palmitic	Capric	Stearic	Oleic
Percentage %	10.4	7.9	18.8	9.9	0.2	1.7	50.3

**Table 12:** Physical characteristics of SonneWarmix RT^TM^.

Property	Color	Oder	Density gm/cm^3^@100 °C	Melting Point	Boiling Point	Flash Point	Kinematic Viscosity@100 °C
Result	Dark brown	Petroleum	0.81	125 °C	>230 °C	>93.4 °C	18 cSt

**Table 13:** Levels of design gyrations according to SHRP-A-407.

Design Traffic (ESALs) × 10^6^	<0.3	0.3 to <3	3 to <10	10 to <30	≥30
N_design_	50	75	100	100	125

**Table 14:** The estimated volumetric properties of the mix.

Property	Result	AASHTO M323 Standards [43]
Estimated Binder (Pb)	4.9%	/
Estimated Voids of Mineral Aggregate (VMA)	14.7	Min. 14%
Estimated Voids Filled with Asphalt (VFA)	72.33	(65–75)%
Estimated Effective Binder (Pbe)	4.94%	/
Dust Proportion	1.32	0.6–1.2
Estimated G_mm_ at N_initial_	87	<89%

**Table 15:** The dosages of recycling agents.

The Aged Binder	WCO% by Weight of Aged RAP Binder *	WEO% by Weight of Aged RAP Binder	DSO% by Weight of Aged RAP Binder	SonneWarmix RT^TM^ by Weight of Total Binder
20% RAP	(0–10%)	(0–10%)	(0–10%)	(0.5–1.0%)
40% RAP	(12.5–17.5%)	(12.5–17.5%)	(12.5–17.5%)	(1.0–1.5%)
60% RAP	(17.5–20%)	(17.5–20%)	(17.5–20%)	(1.5–2.0%)

* The used additive by weight of aged RAP binder = additive% × (% of aged binder by weight of total mix × total binder weight in the mix).

**Table 16:** The volumetric properties of the trial mixtures.

Property	Binder %			
	4.4	4.9	5.4	5.9
G_mb_	2.322	2.384	2.355	2.343
G_mm_	2.465	2.431	2.401	2.378
Air Voids %	6.0	3.6	2.7	2.0
VFA%	57	78	87	93
VMA%	15.7	14.5	14.0	14.8
G_mm_% at N_initial_	85	87	91	94
G_mm_% at N_design_	94	96.5	98	99
G_mm_% at N_max_	95.5	97.5	99	100

**Table 17:** The volumetric properties of the HMA mixture at optimum binder content.

Property	The Result	AASHTO M 323 Standards
Binder optimum content %	4.8	at 4% Air Voids
Air content %	4.0	4.0
VMA %	14.7	Minimum 15%
VFA %	74	(65–75)%
G_mm_ at N_initial_	86.5%	≤89%
G_mm_ at N_design_	96%	96%
G_mm_ at N_max_	97%	≤98%
Dust proportion %	1.1	0.6–1.2

**Table 18:** The added percentages of virgin binder to the recycled mixtures.

RAP Percentage in the Mix	Added Virgin Binder _(by Weight of Total Mix)_	Aged Binder _(by Weight of Total Mix)_	Total Binder Content	Virgin Binder _(by Weight of Total Binder Content)_	Aged Binder * _(by Weight of Total Binder Content)_
20%	4.08%	0.72%	4.8%	85%	15%
40%	3.36%	1.44%		70%	30%
60%	2.64%	2.16%		55%	45%

* The % of aged binder _by weight of of total binder conctent_ = % of aged binder _by weight of total mix_ divided by the % of total binder content.

**Table 19:** Rutting indicator (G*/sin δ) for the unaged samples of the binders’ blend.

Binder Type	G*/sin δ (Unaged)				
	64 °C	70 °C	76 °C	82 °C	88 °C
Virgin binder	3.12	1.62	0.88		
(Aged + virgin) blend of 20% RAP	4.77	2.81	1.11	0.55	
(Aged + virgin) blend of 20% RAP + 10% WCO	3.04	1.54	0.61		
(Aged + virgin) blend of 20% RAP + 10% WEO	3.1	1.6	0.82		
(Aged + virgin) blend of 20% RAP + 10% DSO	2.93	1.48	0.74		
(Aged + virgin) blend of 20% RAP + 0.5% SonneWarmix RT^TM^	3.52	2.03	0.91		
(Aged + virgin) blend of 20% RAP + 1.0% SonneWarmix RT^TM^	3.14	1.91	0.9		
(Aged + virgin) blend of 40% RAP	5.28	4.12	2.42	1.12	0.56
(Aged + virgin) blend of 40% RAP + 12.5% WCO	3.47	1.96	0.91		
(Aged + virgin) blend of 40% RAP + 17.5% WCO	2.95	1.5	0.6		
(Aged + virgin) blend of 40% RAP + 12.5% WEO	3.25	1.73	0.87		
(Aged + virgin) blend of 40% RAP + 17.5% WEO	3.15	1.64	0.85		
(Aged + virgin) blend of 40% RAP + 12.5% DSO	3.2	1.63	0.83		
(Aged + virgin) blend of 40% RAP + 17.5% DSO	2.83	1.24	0.6		
(Aged + virgin) blend of 40% RAP + 1.0% SonneWarmix RT^TM^	3.15	1.65	0.89		
(Aged + virgin) blend of 40% RAP + 1.5% SonneWarmix RT^TM^	2.78	1.41	0.71		
(Aged + virgin) blend of 60% RAP	6.46	5.21	3.81	2.15	0.98
(Aged + virgin) blend of 60% RAP + 17.5% WCO	3.92	2.14	1.15	0.58	
(Aged + virgin) blend of 60% RAP + 20% WCO	2.97	1.54	0.61		
(Aged + virgin) blend of 60% RAP + 17.5% WEO	3.49	2	0.95		
(Aged + virgin) blend of 60% RAP + 20% WEO	2.59	1.27	0.6		
(Aged + virgin) blend of 60% RAP + 17.5% DSO	4.33	2.08	1.05	0.51	
(Aged + virgin) blend of 60% RAP + 20% DSO	2.8	1.55	0.63		
(Aged + virgin) blend of 60% RAP + 1.5% SonneWarmix RT^TM^	5.02	2.04	1.02	0.50	
(Aged + virgin) blend of 60% RAP + 2.0% SonneWarmix RT^TM^	3.2	1.61	0.87		

**Table 20:** Rutting indicator (G*/sin δ) for the RTFO aged samples of the developed binder.

Binder Type	G*/sin δ (RTFO)				
	64 °C	70 °C	76 °C	82 °C	88 °C
Virgin binder	5.14	1.94			
(Aged + virgin) blend of 20% RAP	6.24	3.02	1.84		
(Aged + virgin) blend of 20% RAP + 10% WCO	5.02	1.83			
(Aged + virgin) blend of 20% RAP + 10% WEO	5.11	1.97			
(Aged + virgin) blend of 20% RAP + 10% DSO	4.87	1.76			
(Aged + virgin) blend of 20% RAP + 0.5% SonneWarmix RT^TM^	5.46	2.24	1.89		
(Aged + virgin) blend of 20% RAP + 1.0% SonneWarmix RT^TM^	5.35	2.11	1.86		
(Aged + virgin) blend of 40% RAP	7.34	4.22	2.65	1.94	
(Aged + virgin) blend of 40% RAP + 12.5% WCO	5.38	2.17	1.86		
(Aged + virgin) blend of 40% RAP + 17.5% WCO	4.89	1.79			
(Aged + virgin) blend of 40% RAP + 12.5% WEO	5.35	2.2	1.87		
(Aged + virgin) blend of 40% RAP + 17.5% WEO	5.17	1.96			
(Aged + virgin) blend of 40% RAP + 12.5% DSO	5.2	2			
(Aged + virgin) blend of 40% RAP + 17.5% DSO	4.65	1.54			
(Aged + virgin) blend of 40% RAP + 1.0% SonneWarmix RT^TM^	5.19	1.98			
(Aged + virgin) blend of 40% RAP + 1.5% SonneWarmix RT^TM^	4.74	1.63			
(Aged + virgin) blend of 60% RAP	7.88	5.11	4.56	2.77	2.15
(Aged + virgin) blend of 60% RAP + 17.5% WCO	5.22	2.86	1.96		
(Aged + virgin) blend of 60% RAP + 20% WCO	4.91	1.83			
(Aged + virgin) blend of 60% RAP + 17.5% WEO	5.4	2.3	1.89		
(Aged + virgin) blend of 60% RAP + 20% WEO	5.0	1.57			
(Aged + virgin) blend of 60% RAP + 17.5% DSO	7.67	5.36	1.49		
(Aged + virgin) blend of 60% RAP + 20% DSO	4.81	2.0			
(Aged + virgin) blend of 60% RAP + 1.5% SonneWarmix RT^TM^	5.77	2.94	1.98		
(Aged + virgin) blend of 60% RAP + 2.0% SonneWarmix RT^TM^	5.19	1.97			

**Table 21:** Rutting depth for the control and rejuvenated HMA average mixtures.

Mixture Type	Rut Depth (mm)			
	2500 Cycles	5000 Cycles	7500 Cycles	10,000 Cycles
Control HMA Mix	4.13	6.34	7.88	9.12
HMA of 20% RAP	5.51	8.66	9.81	11.79
HMA of 20% RAP + 10% WCO	4.21	6.51	7.92	9.4
HMA of 20% RAP + 10% WEO	4.15	6.37	7.9	9.17
HMA of 20% RAP + 10% DSO	5.32	8.34	9.7	11.6
HMA of 20% RAP + 0.5% SonneWarmix RT^TM^	3.75	5.13	6.71	7.9
HMA of 20% RAP + 1.0% SonneWarmix RT^TM^	3.82	5.77	7.56	8.02
HMA of 40% RAP	6.15	8.95	10.48	12.20
HMA of 40% RAP + 12.5% WCO	3.8	5.64	7.32	8
HMA of 40% RAP + 17.5% WCO	5.3	8.3	9.7	11.58
HMA of 40% RAP + 12.5% WEO	3.6	5.57	6.8	7.77
HMA of 40% RAP + 17.5% WEO	4.1	6.3	7.5	9
HMA of 40% RAP + 12.5% DSO	4	6.1	5.5	9
HMA of 40% RAP + 17.5% DSO	5.5	8.7	10.1	11.7
HMA of 40% RAP+ 1.0% SonneWarmix RT^TM^	4.1	6.4	7.5	9.1
HMA of 40% RAP + 1.5% SonneWarmix RT^TM^	5.8	8.6	10.1	11.7
HMA of 60% RAP	6.95	9.28	11.70	13.11
HMA of 60% RAP + 17.5% WCO	3	5.1	6.24	7.52
HMA of 60% RAP + 20% WCO	5.3	8.3	9.7	11.58
HMA of 60% RAP + 17.5% WEO	3.2	5.35	6.3	7.3
HMA of 60% RAP + 20% WEO	5.3	8.2	9.5	11.24
HMA of 60% RAP + 17.5% DSO	3.2	4.3	5	6.2
HMA of 60% RAP + 20% DSO	5.5	8.5	9.3	10.1
HMA of 60% RAP+ 1.5% SonneWarmix RT^TM^	2.9	4.1	4.9	6
HMA of 60% RAP+2.0% SonneWarmix RT^TM^	4.1	6.3	7.86	9.12

**Table 22:** The optimum percentages of the used rejuvenators.

Rejuvenator Type	Optimum Percentage		
	@ 20% RAP	@ 40% RAP	@ 60% RAP
WCO _by weight of the aged RAP binder_	10%	12.5%	17.5%
WEO _by weight of the aged RAP binder_	10%	12.5–17.5%	17.5%
DSO _by weight of the aged RAP binder_	<10%	12.5%	17.5%
SonneWarmix RT^TM^_by weight of the total binder_	0.5–1.0%	1.0%	1.5–2.0%

## Data Availability

Not applicable.
